# Heterostructure and *Q*-factor engineering for low-threshold and persistent nanowire lasing

**DOI:** 10.1038/s41377-020-0279-y

**Published:** 2020-03-17

**Authors:** Stefan Skalsky, Yunyan Zhang, Juan Arturo Alanis, H. Aruni Fonseka, Ana M. Sanchez, Huiyun Liu, Patrick Parkinson

**Affiliations:** 1 Department of Physics and Astronomy and The Photon Science Institute, The University of Manchester, Manchester, M13 9PL UK; 2 Department of Electronic and Electrical Engineering, University College London, London, WC1E 7JE UK; 3 Department of Physics, University of Warwick, Coventry, CV4 7AL UK

**Keywords:** Semiconductor lasers, Fluorescence spectroscopy, Nanowires

## Abstract

Continuous room temperature nanowire lasing from silicon-integrated optoelectronic elements requires careful optimisation of both the lasing cavity *Q*-factor and population inversion conditions. We apply time-gated optical interferometry to the lasing emission from high-quality GaAsP/GaAs quantum well nanowire laser structures, revealing high *Q*-factors of 1250 ± 90 corresponding to end-facet reflectivities of *R* = 0.73 ± 0.02. By using optimised direct–indirect band alignment in the active region, we demonstrate a well-refilling mechanism providing a quasi-four-level system leading to multi-nanosecond lasing and record low room temperature lasing thresholds (~6 μJ cm^−2^ pulse^−1^) for III–V nanowire lasers. Our findings demonstrate a highly promising new route towards continuously operating silicon-integrated nanolaser elements.

## Lasers: Record-breaking lasing nanowires

Persistent short pulses of near-infrared laser light can be produced at record low thresholds using tiny nanowire lasers, suitable for small-scale optoelectronic devices, including optical computing chips. The technology has been developed and demonstrated by researchers in the UK, led by Patrick Parkinson at the University of Manchester. Their system is based on semiconducting nanowires composed of gallium arsenide phosphide and gallium arsenide. It combines both key functions of a laser in one material, as the gain medium that amplifies the light emission and the cavity medium for containing and manipulating the light. The researchers engineered the precise behavior of the nanowires to demonstrate highly optimized nano-lasing performance at room temperature lasing thresholds. Their work addresses and overcomes significant challenges that have been limiting developments in this promising field.

## Introduction

The desire to reduce the footprint of laser systems for enabling low-power compact nanophotonic applications such as on-chip optical computing^1,2^ and telecommunications^3^ has inspired extensive research with respect to the development and characterisation of optoelectronic nanostructures^4^. Among the most promising candidates are semiconductor nanowires (NWs), primarily due to their dual function as both the gain material and cavity^5–7^, their ease of integration with silicon^8^ and the possibility of both radial and axial engineering^9,10^. The recent growth of NW laser cavities with embedded quantum wells provides further advantages from quantum confinement, including emission tunability^11^, lower lasing thresholds^8,12^ and narrower emission linewidths^13,14^. While developments in this field are promising, the full characterisation of nanolaser (NL) performance and specifically the cavity quality is challenging because techniques conventionally used for macroscopic laser characterisation and for end-facet reflectivity measurements in particular are highly technically demanding when used for NW applications^15^. These technical demands have led to the development of many advanced modelling approaches^16^; however, experimental studies such as those employing time-resolved techniques revealing information on the underlying charge carrier and photon dynamics have been scarce^17^ and are urgently needed to separate the influence of the gain material and cavity design on the functional performance and thus enable more focused fabrication optimisation.

**Fig. 1:**
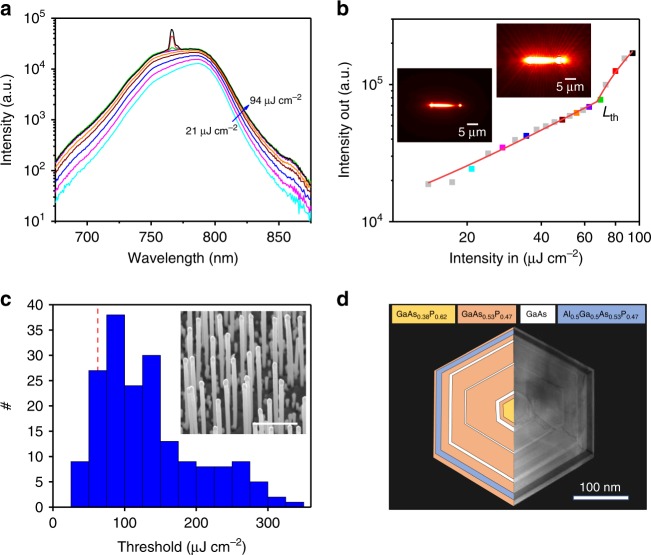
Characterisation of a typical NW laser. **a** Power-dependent emission spectra on a log scale, showing PL clamping at the onset of lasing, followed by narrow single-mode emission. **b** The light intensity-in vs. intensity-out plot corresponding to the same wire/data in **a** on a log–log scale (with the output measured within a narrow spectral range of *λ*_Las_) and fit with the indicated threshold position (*L*_th_). Data points are colour matched to the corresponding spectra in **a**, and the grey data points indicate additional powers not included in **a**. The two insets show emission images of the same NW, taken (left) just below and (right) above threshold, showing the emergence of spatially coherent emission from the end facets. **c** A histogram of the lasing thresholds for all 270 wires, with the example NL in **a**, **b** indicated by a vertical red dashed line. The inset shows an SEM image of the NWs after MBE growth, with the scale bar representing 5 μm in length. **d** TEM image of the NW cross-section (right) and schematic illustrating the structure (left)

In this work, we address this challenge by using a time-gated interferometric method (i-TCSPC [interferometric time-correlated single-photon counting]) to study the simultaneous temporal and spectrally resolved emission dynamics of molecular beam epitaxy (MBE)-grown near-IR GaAsP*/*GaAs-based quantum well NW lasers at room temperature. The wires consist of a GaAs_1 − *x*_P_*x*_ core and barriers with three active coaxial GaAs quantum wells, where *x* = 0.62 and *x* = 0.47 in the core and barriers, respectively (see Fig. 1d for the schematic and transmission electron microscopy [TEM] image of structure), providing deep carrier confinement as well as strong overlap of the cavity modes with the gain media. An Al_0.5_Ga_0.5_As_0.53_P_0.47_ outer protective layer is employed to protect the NW from oxidation effects. These NLs have potential advantages over their more established AlGaAs/GaAs counterparts^18^, including having highly strained quantum wells with deep confinement potential^14^ and a core-absorption-free band profile^19^, leading to external thresholds as low as 20 μJ cm^−2^ pulse^−1^ at 6 K.

Here, we demonstrate the first direct measurements of the emission coherence lengths and end-facet reflectivities for NW lasers. Through photoluminescence excitation (PLE) measurements and by modelling the time-resolved emission spectra, we reveal the underlying carrier dynamics enabling a unique nanolasing mechanism for these wires. By engineering the radial bandstructure alignment, we demonstrate highly optimised nanolasing performance.

## Results

### Large-scale power-dependent μ-PL characterisation

Excitation fluence-dependent PL characterisation was carried out on 270 isolated wires^20–22^, using a 620 nm sub-picosecond excitation pulse (details are provided in the Methods section). A typical measurement is shown in Fig. 1. The lasing thresholds (*L*_th_) are conventionally determined from the positions of the kinks in the measured intensity-in vs. intensity-out plots on a log–log scale, where excitation above the threshold leads to the emergence of amplified spontaneous emission followed by coherent lasing emission. However, many of the NLs studied did not demonstrate clear kinks in their threshold curves despite displaying other characteristics of lasing, including PL clamping, narrow lasing modes and spatially coherent emission within the spectra and imaging. This finding suggests that these lasers may have high spontaneous emission factors (*β*)^23^, which reflects the optical efficiency of the structures. Reports in the literature for multiple quantum well NLs have modelled *β* values of ~0.03 at room temperature^13^. The small kink in the light-in light-out curve (shown in Supplementary Fig. S3) indicates that our wires are comparable or better than those previously demonstrated^13^. We attribute this behaviour to a high-quality (Q) cavity design with strong overlap of the cavity modes and the gain media.

To determine the lasing threshold, the photoemission was integrated within a close spectral range of the lasing modes, under which circumstances clearer kinks are observed as shown in Fig. 1b, with a histogram of all measured lasing thresholds shown in Fig. 1c. The power-dependent spectra (Fig. 1a) show broad spontaneous emission below this threshold, which slightly blueshifts (~5 nm) and broadens upon increasing pump fluence due to bandfilling effects. When the lasing threshold is reached, a narrow lasing peak (*λ*_Las_ ≈ 770 nm) emerges, superposed on the spontaneous emission background. Upon increased excitation fluence, there is evident PL clamping and channelling of energy into the lasing mode, which begins to dominate the spectra. Similar to the NW data shown in Fig. 1, many of the 270 NLs studied demonstrate single-mode emission, which is crucial for limiting group velocity dispersion and temporal pulse broadening for data communications applications^24^. The NLs were found to have a high fractional lasing yield, with 87% of those studied found to lase before the onset of thermally induced degradation. Typical lasing thresholds of ~100 μJ cm^−2^ pulse^−1^ were measured under a 620 nm excitation with a champion threshold of 32 μJ cm^−2^ pulse^−1^, which is competitive with the lowest reported thresholds for III–V NW lasers^20^. Additional spectra and threshold curves for other NW lasers from this growth are shown in the Supporting Information. Furthermore, these wires were found to be stable under high excitation intensity, with measured damage thresholds of over 2000 μJ cm^−2^ pulse^−1^ (see Supplementary Information for more details). Together, the low threshold and high stability are further indicative of a high-Q cavity. Notably, the emission from optical images often appears with a spatially non-uniform distribution along the NW axis (see Fig. 1b inset). It is expected that lasing emission will be effectively waveguided and emitted from the end facets, however, residual spontaneous emission will continue to be emitted after the lasing transient ends, from the body of the wire. This emission is often inhomogeneous, as stacking faults can concentrate at one end^14^ due to the vapour–liquid–solid growth mechanism. While this problem associated with the presence of such dislocations is commonly reported in NW structures^25^, it does not appear to affect the lasing performance for the presented wires, likely due to the reduced optical absorption in these structures at the lasing wavelength.

### End-facet reflectivity

**Fig. 2:**
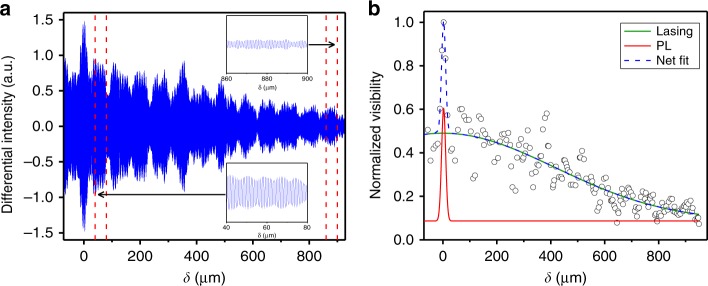
**a** A time-gated interferogram integrated over time 0 <*τ* < 1 ns after excitation. The two regions are expanded for clarity, showing coherence over hundreds of microns. The slow beating is indicative of two (unresolved) neighbouring lasing peaks, possibly arising from the broken degeneracy of the orthogonal transverse modes^13,14^. **b** The fitted visibility is shown for the data in **a**, indicating the spontaneous (red line) and lasing (green line) contributions to the fit (dashed blue line)

The coherence length (*L*_coh_) of a laser is related to the number of round trips photons make in the cavity and is thus also related to the photon cavity lifetime (*τ*_p_), end-facet reflectivity (*R*) and *Q*-factor. The direct measurement of these factors can be challenging for the NW architecture using conventional approaches. In this study, the above-threshold lasing emission from the wires using the i-TCSPC technique is investigated, which combines Fourier transform spectroscopy and traditional TCSPC in a two-channel Michelson interferometer. This technique is used to obtain ultrasensitive measurements of both temporal and spectrally resolved emission and is fibre coupled in a quasi-confocal microscopy arrangement (see Supplementary Information for more details). The i-TCSPC directly provides time-gated interferometry, also known as the *g*^(1)^ correlation, as shown for a selected NW laser in Fig. 2. The interferogram for the emission following above-threshold excitation (1.4 *L*_th_) is shown in Fig. 2a. We use the procedure of Liu et al.^26^, fitting the interference visibility ($\frac{{I_{\mathrm{max}}\left( \delta \right) - I_{\mathrm{min}}\left( \delta \right)}}{{I_{\mathrm{max}}\left( \delta \right) + I_{\mathrm{min}}\left( \delta \right)}}$), where *δ* is the optical path length difference between the two arms of the interferometer, with the sum of the Gaussian PL and lasing components (shown in Fig. 2b), to determine a NL coherence length of 980 ± 70 μm (the full-width at half-maximum of the lasing visibility). While Liu employed this method in the study of nano-cavities based on two-dimensional (2D) transition metal dichalcogenide materials, it is equally applicable to the measurement of the coherence length for a semiconductor NW laser. The determined value is ~70× the NW physical length (14.5 μm) and corresponds to a photon cavity lifetime *τ*_p_ = 0.51 ± 0.04 ps, a mean end-facet reflectivity *R* = 0.73 ± 0.02 and a cavity *Q*-factor, *Q* = 1250 ± 90 (see Supplementary Information for details of the experimental procedure and calculations). Such high resonator quality levels are comparable to those demonstrated by photonic crystals^27^ and plasmonic NL structures^28^. Conventionally, laser *Q*-factors are determined directly from the ratio of the lasing frequency and bandwidth measured for time-integrated emission^29^; however, this factor can be less reliable for pulsed systems with dynamic behaviour, such as for NLs.

Notably, our calculation of the cavity properties assumes no distributed losses within the cavity (i.e. no absorption by the core/barriers). While the core and barriers are expected to have a higher bandgap than the emitted light energy, our values for the reflectivity and *Q*-factor are strictly a minimum. Classical Fresnel reflectivity calculations $(( {\frac{{n - 1}}{{n + 1}}})^2)$ are known to break down in nano-cavity regimes where the diameter is smaller than the wavelength; this yields a reflectivity enhancement^30^. Using a refractive index of 3.4 (for GaAsP at *λ*_Las_ = 780 nm^31^), we determined an increase in the end-facet reflectivity of more than 2× over the naive Fresnel reflectivity (0.30); these values are comparable to those obtained using simulation methods^13^. As such, it is clear that the high reflectivity in these nano-cavities contributes to the low threshold values measured for the NLs based on them. This result is highly promising for the development of electrically pumped NLs that can be integrated onto a silicon platform.

### Excitation-energy-dependent emission

A compromise is often observed between the excitation energy and lasing threshold in NLs; at excitation energies close to the bandedge, the external absorption cross-section can be low due to geometrical effects^32^, while at energies far above the bandedge, thermalisation effects after excitation can lead to accelerated thermal degradation. For core/shell NWs, this effect is further complicated by multiple absorption and carrier transfer routes. For the GaAsP/GaAs NWs studied, the core and barrier material stoichiometries lead to a *Γ*-point close to the direct–indirect transition^33^, which provides an additional design opportunity. To gain insight into the effect of the band-structure alignment, the effective wavelength-dependent absorption (and subsequent coupling to the emission channel) was investigated for seven NWs via low-fluence PLE measurements along with an excitation-wavelength-dependent lasing threshold measurement for a single NW (see Methods for full details). PLE measurements were carried out for wires deposited on quartz substrates, while other measurements were carried out on silicon. The individual wires studied in the threshold dependence study were different from those studied via PLE; however, all seven NWs studied using PLE showed similar spectra (see Supplementary Information).

**Fig. 3:**
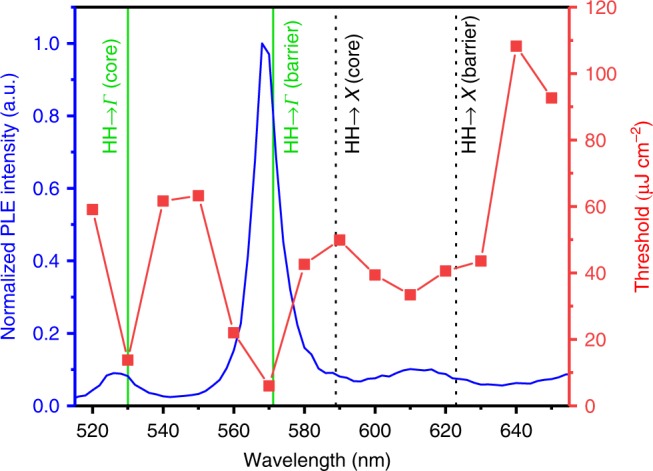
Wavelength-dependent PL excitation spectra (blue) and corresponding lasing thresholds (red). Calculated direct (solid green lines) and indirect (dashed black lines) absorption transitions are indicated

Figure 3 shows a measured PLE spectrum consisting of multiple absorption features. Lasing threshold measurements were performed as a function of the excitation wavelength over the same range; intriguingly, the maxima in the PLE signal correspond to the minima in the lasing thresholds, indicating that these absorption peaks not only increase the PL but also provide a route to the generation of a population inversion in the quantum well. For both studies, this is unexpected; previous studies have shown a monotonically increasing PLE signal with increasing energy above the emission edge^34^ or a broad resonance^32^. While oscillations due to exciton–phonon coupling have been observed to cause resonances in the 2D density of states^35^, we do not anticipate this effect at room temperature. It is more likely that the resonance-like features are a result of inter-band absorption in the single NWs, which is a product of the band-structure and optical resonances^36,37^. For the large-diameter NWs studied, optical resonance effects are less strong; furthermore, unpolarised excitation and detection were used in the PLE study, further reducing the effect of any resonances that are known to be strongly polarisation dependent. The shape of the PLE and threshold spectra must therefore arise from the underlying bandstructure of the NW.

Computational modelling of the bandstructure was carried out in the Nextnano software^38,39^ (see Methods and Supplementary Information), and the predicted room temperature optical transitions were calculated. The overlaid vertical lines in Fig. 3 show the calculated direct and indirect absorption transitions. Good agreement between the simulated transitions and the experimental data was observed for phosphorous concentrations (*x*) of the GaAs_1 − *x*_P_*x*_ core and barriers of 0.69 and 0.54, respectively. These values are ~10% higher than the nominal values reported previously^14^. This result is possibly due to variations in the phosphorous concentration across the growth substrate due to the known difficulty in controlling the phosphorous concentration via MBE growth^40^ or inaccuracies arising from the modelling of the strain.

Excitation with a photon energy below the lowest-lying barrier transition (*HH* → *X*) is associated with a decrease in the PLE signal and an increase in the lasing threshold, showing that barrier absorption contributes to quantum well population inversion via carrier transfer. This finding is striking for two reasons. The lowest transition is predicted to be indirect, so it requires the scattering of electrons from the *X*-band of the barrier to the confined well state. Additionally, the material quality must be sufficiently high that injection into the well is efficient when compared with carrier trapping at defects in the barrier. Importantly, at a 570 nm excitation, a large absorption resonance and low corresponding lasing threshold of 6 μJ cm^2^ pulse^−1^ is observed (see Supplementary Information for the corresponding power-dependent spectra and intensity-in vs. intensity-out plots). We attribute this feature to a strong, higher-lying direct absorption in the barrier (*HH* → *Γ*) leading to high carrier densities, followed by efficient carrier transfer into the confined well states. This threshold is significantly lower than the previously reported room temperature lasing thresholds for near-infrared III–V NW lasers^20,22,41^.

The resonance features in the PLE and laser-threshold study are narrow, with a full-width at half-maximum of ~40 meV. At present, the origin of the shape of this feature is unclear, although the width is similar in size to both the optical phonon energy in GaP^42^ and the strain-induced light-hole heavy-hole splitting in our material (as determined from the simulation, as shown in the Supplementary Information).

### Persistent lasing emission

To probe the carrier transfer dynamics in these heterostructured materials, time- and energy-resolved spectroscopy was performed on ten wires at a series of excitation powers (below, around and above the threshold). Time-resolved emission spectroscopy is of great use to understand recombination dynamics and can be related to processes such as bandgap renormalisation, interfacial recombination^43^ and energy transfer^44^. i-TCSPC measurements were performed with a 620 nm excitation (chosen to be close the barrier energy without incurring significant heating effects). All wires were found to display very similar behaviour demonstrating reliability; we restrict the following discussion to a single typical NL.

**Fig. 4:**
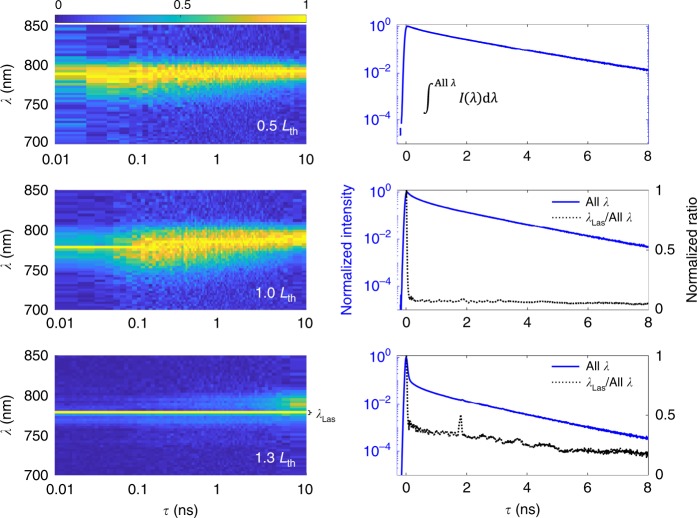
Normalised time-resolved PL maps (left) and emission decays (right) for the selected NL. The blue line is the emission integrated across the whole emission band, while the dotted black line (where relevant) shows the normalised ratio of lasing emission to the total emission on a linear scale (right *y*-axis). The measurements are taken following below threshold (0.5 *L*_th_, top), close to threshold (1.0 *L*_th_, middle) and above-threshold (1.3 *L*_th_, bottom) excitation. The transient increase in the ratio observed for above threshold excitation close to 1.8 ns is associated with a reflection in the system. A common colour bar is shown for the three maps

Figure 4 shows time-resolved PL maps and time decays for the NL following below threshold (0.5 *L*_th_), at threshold (1.0 *L*_th_) and above-threshold excitation (1.3 *L*_th_) where *L*_th_ is the threshold fluence. Below the threshold, pure broad spontaneous emission occurs at all times after excitation (*τ*), which slightly redshifts and narrows as the carrier density drops, and carriers relax to the bandedge. The corresponding time decay is nearly mono-exponential with a time constant *τ*_spont_ = 2 ns. Similar PL decay rates have been previously reported in GaAs/GaAsP NW structures^45^. At a threshold fluence, a narrow lasing peak emerges at early times (*τ* < 100 ps), which decays and is replaced by broad spontaneous emission. The corresponding time decay becomes bi-exponential with an additional fast component that we attribute to stimulated emission. By monitoring the emission intensity at the lasing wavelength (marked as *λ*_Las_), the stimulated component is seen to decay with a time constant of ~30 ps, which corresponds to the resolution of our system (the instrument resolution is measured to be 33 ps half-width at half-maximum).

When pumped above the threshold, the lasing peak emission dominates at all *τ*. The emergence of a long-lived lasing component for the above-threshold excitation is striking; both the excitation pulse and cavity lifetime are below 1 ps. To probe this behaviour further, time-gated emission spectra were fitted with the sum of a PL component (approximated as a Gaussian with variable centre energy *E*_PL_, width *σ*_PL_ and height *y*_PL_) and a lasing component (a Gaussian with fixed centre energy *E*_Las_ and width *σ*_Las,_ and variable height *y*_Las_):

1 $$\begin{array}{l}I\left( E \right) = y_{\mathrm{PL}}\exp \left( {\frac{{ - \left( {E - E_{\mathrm{PL}}} \right)^2}}{{2\sigma _{\mathrm{PL}}^2}}} \right)\\ +\; y_{\mathrm{Las}}\exp \left( {\frac{{ - \left( {E - E_{\mathrm{Las}}} \right)^2}}{{2\sigma _{\mathrm{Las}}^2}}} \right)\end{array}$$

**Fig. 5:**
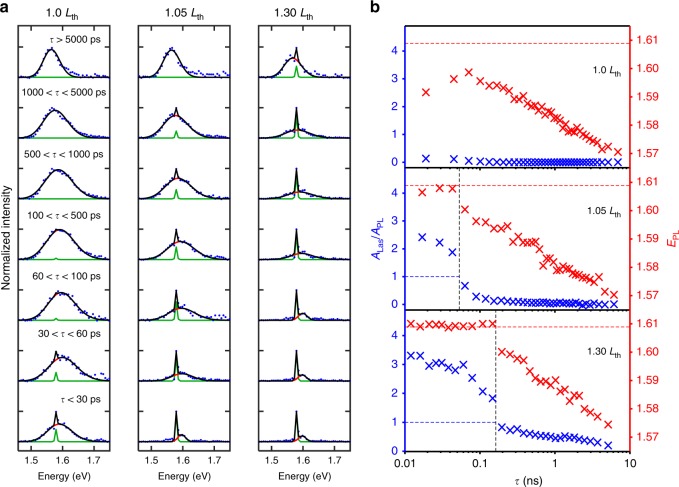
**a** Spectra extracted from the time-gated emission study for each excitation regime are shown (blue dots) for selected delays, with a fit as described in the main text (black line), with the PL component (red line) and lasing component (green line) indicated. **b** The peak PL component energy and relative lasing/PL intensity ratio are shown as a function of time after excitation for each excitation regime. The vertical black lines indicate where *A*_Las_*/A*_PL_ drops below 1 (shown as a blue horizontal dashed line), while the horizontal red dashed lines indicate the approximate energy at which the PL emission is capped

Time-gated spectra and fits are shown in Fig. 5a. The evolution of the relative emission contained in the lasing mode compared to the PL (given by the ratio between areas under the Gaussian fits (*A*_Las_/*A*_PL_)) and the peak PL energy (*E*_PL_) were extracted as a function of time for each excitation fluence, that is, at threshold (1.0 *L*_th_) and above threshold (1.3 *L*_th_) as well as an intermediate power (1.05 *L*_th_), as shown in Fig. 5b. The time bins were chosen such that an equal number of photons fall within each bin to optimise the signal-to-noise ratio and thus are more closely spaced at early *τ* values where the intensity is higher.

At the threshold, the ratio of light emitted into the lasing component to that in the PL component is 0.15, which rapidly decays to zero within ~100 ps. In the 1.05 *L*_th_ regime, the intensity of light in the lasing component is ~2.5 times greater than the PL at early times. The lasing component drops rapidly, losing its dominance within ~100 ps, after which it decays more slowly and does not fully disappear until >5 ns. Under the highest excitation fluence, the initial ratio of lasing to the PL exceeds 3, but intriguingly, the early ratio decay is delayed, with lasing remaining dominant over the PL until close to 200 ps. There is again a slow secondary decay that does not fully deplete within the full 10 ns.

In all cases, the reduction in the relative lasing magnitude is associated with a PL redshift. This redshift is attributed to a drop in the carrier density after initial band filling within the quantum well. Significantly, this redshift is delayed in the highest excitation regime, which strongly indicates that both the roll-off in lasing and redshifting in the PL arise from the same physical mechanism.

After excitation at 620 nm, photocarriers are excited into both the well and the barrier regions^14^. Under high excitation fluence conditions, the electron well states responsible for the gain—the transition from the lowest confined electron state to the highest confined hole state—can be completely filled to the confinement energy of the well, leading to a capping of the peak PL energy at ~1.609 eV (Fig. 5b). Multiple hole states are confined and therefore will not reach saturation until a much higher (~6×) carrier density.

We attribute the strong excitation dependence of *A*_Las_/*A*_PL_ and the corresponding long-lived lasing at 1.3 *L*_th_ to continuous electron transfer from the GaAsP barriers into the active GaAs quantum wells, effectively refilling the well and extending the population inversion. At the highest fluence used, the PL emission remains at an energy of 1.609 eV for over 100 ps; this is associated with carrier transfer as a process that supports both PL and lasing. Evidence for carriers absorbed into the barrier contributing to lasing is seen in Fig. 3, where a decrease in the lasing thresholds is observed for excitations at energies higher than the barrier absorption edge. Calculations of the radial bandstructure show that the indirect *X*-valley is nearly isoenergetic throughout the radius of the wire (within *k*_B_*T*, see Supplementary Information), which increases the likelihood of radial carrier diffusion.

## Discussion

**Fig. 6:**
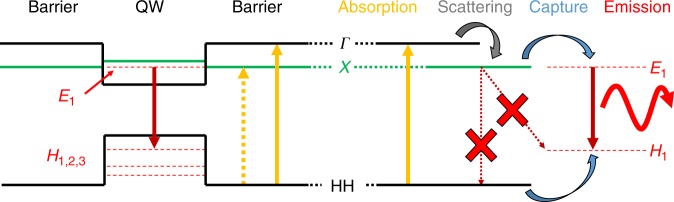
Schematic of the energy processes occurring following optical excitation (left) as a function of the radial position and (right) as a function of time. The direct transitions are shown with solid vertical lines, and the indirect transitions are shown with dashed lines. The optical excitation of carriers into the barrier is followed by rapid (picosecond) scattering, both directly into the confined states in the well and into the *X*-valley in the barrier. This excitation is followed by slow (nanosecond) electron scattering into the well from the *X*-valley of the barrier, which is near isoenergetic with the *X*-valley energy. Direct carrier recombination from the *X* to the *HH* band is suppressed. In the case of high excitation density, the electron states in the well will become filled, and electrons will remain stored in the barrier *X*-valley

A schematic of our proposed energy mechanism is shown in Fig. 6. The generation of conduction-band electrons in the quantum well can occur through direct absorption or transfer from the *X* or *Γ* states of the barrier. Of these, the latter is associated with the strongest optical absorption due to the relatively larger volume of barrier material with respect to core material and the symmetry of the transition. Following absorption into the *Γ*-band, rapid (picosecond timescale) inter-valley scattering will result in an electron population in the *X*-valley^46^. It is possible that carrier capture into the quantum well electron states occurs directly from the *Γ*-band, but this process is unlikely to be competitive at distances farther than a few nanometres from the barrier–well interface due to the short time that electrons remain in this band. Holes created in the barrier can be captured efficiently to at least three confined hole states in the well (determined by modelling); however, electrons have a single level to transfer to, which gives rise to the possibility of bandfilling at which time no further carrier capture can occur. Electrons will remain in the *X*-band until recombination, trapping, or the freeing of an available state in the *E*_*1*_-band of the quantum well to transfer into. However, recombination is suppressed, both by the symmetry of the transition and the spatial separation of electrons (in the barrier) and holes (in the well), which provides the possibility for prolonged (nanosecond scale) well refilling from the *X*-band of the barrier under sufficiently high excitation conditions. The existence of this process is strongly indicative of high-quality barrier material with low defect-assisted recombination rates. The mechanism has a desirable resemblance to a four-level laser system with barriers acting analogously to the meta-stable lasing levels.

Lasers based on semiconductor NWs allow for the ease of hetero-integration due to the relaxed strain lattice-matching requirements^47^. For the wires presented, this effect provides an opportunity for band-structure alignment, giving rise to the unexpected advantage of a quasi-four-level lasing system by exploiting indirect bands as the holding states. The very high material and cavity quality enables fundamental energy dynamics to be observed. It is expected that this NL architecture may be further optimised through the number and position of the quantum wells, adding to the overall gain, as has been previously demonstrated^13^. The lasing threshold of 6 μJ cm^−2^ pulse^−1^ is evidence that this platform has the capability to offer continuous operation room temperature lasing, from a material system that can be hetero-integrated on to silicon^14^, with a wavelength that can be tuned using the quantum well width as a parameter.

In conclusion, we report the room temperature characterisation of GaAsP/GaAs core/shell quantum well near-IR NW lasers using both power dependence measurements and a time-resolved interferometric methodology (i-TCSPC). The NLs demonstrate reliable and high-quality performance with an operational yield of over 85%, a high damage threshold and record low lasing thresholds of ~6 μJ cm^−2^ pulse^−1^. Time-resolved interferometry revealed high-Q cavities (1250 ± 90) and corresponding super-Fresnel end-facet reflectivities (0.73 ± 0.02), which contribute to the high performance in these wires. Under high-intensity excitation, unexpectedly long-lasing times on the order of nanoseconds are observed, which is attributed to a well-refilling effect arising from carrier transfer from the high-quality indirect-gap barriers. These findings of high performance along with controllable carrier dynamics demonstrate great promise for these NL structures for various future nanophotonic applications, including the potential for electrically pumped, silicon-integrated, continuous lasing applications.

## Materials and methods

### NW fabrication and transfer to substrates

The NW structures were grown using MBE directly on silicon, following a previously described recipe^14^ (SEM and TEM imagery are shown in Fig. 1c, d, respectively). NWs are formed with a zinc blende crystal structure of ~10 μm in length and ~400 nm in diameter, consisting of GaAs_1 *−* *x*_P_*x*_ cores and barriers with three active coaxial GaAs quantum wells (varying between 3.5 and 10 nm thick), where *x* = 0.47 and 0.62 in the outer barrier and cores, respectively (as determined using EDX^14^). The post-growth measurements in this study were all carried out at room temperature with the NWs removed from their growth medium and redeposited via ultrasonication onto quartz substrates for the PLE study and on silicon substrates for all other measurements.

### Power-dependent μ-PL measurements

A home-built μ-PL set-up was used to find the NWs using machine vision and carry out PL and power-dependent PL measurements, as previously described^20–22^. An amplified Ti:sapphire excitation laser pulse was coupled to an optical parametric amplifier (OPA) providing pulses at 620 nm, with a 200 fs duration and 250 kHz repetition rate. The incident fluence was controlled using a neutral density filter wheel and continuously measured using a calibrated silicon photodiode. A defocusing lens was used to ensure a large-area excitation (~20 μm diameter, measured using a calibrated optical camera), which uniformly excites the NL, and the emission was collected from one end-facet using fibre-confocal microscopy. The measured calibrated powers were converted to energy densities per pulse by accounting for the repetition rate and beam area. For time-integrated measurements, the microscope emission was fibre coupled to a cooled dispersive spectrometer.

### PLE measurements

PLE measurements were carried out on seven NLs on quartz substrates. A Fianium supercontinuum laser coupled to a monochromator was used as the excitation source, and a cooled dispersive fibre-coupled spectrometer was used for detection, with the signal integrated over the range 650–850 nm (corresponding to the NW PL region). Excitation-wavelength steps of 5 nm were employed for six of the NW measurements along with a high-resolution measurement (2 nm steps) for the other NWs (shown in Fig. 3). The system was corrected for background and excitation-energy variation by monitoring the excitation intensity with a silicon diode. See Supplementary Information for the results of all seven NW PLE measurements.

### Excitation-wavelength threshold dependence measurements

To vary the excitation wavelength, the OPA wavelength was sequentially tuned and optimised. Power dependence measurements were carried out for a single NL with excitation energies between 520 and 650 nm with 10 nm steps; the power was monitored by a calibrated silicon photodiode, which was corrected for spectral response. Thresholds at each wavelength were deduced from the kink position in the intensity-in vs. intensity-out plots.

### Computation modelling in Nextnano

The bandstructures and resulting optical properties of the NW heterostructures were calculated with Nextnano software using a 1D model of pseudomorphically strained zinc blende GaAsP/GaAs core, quantum well and barrier regions. The modelled heterostructures consisted of 5 nm GaAs quantum wells with a GaAs_0.31_P_0.69_ core (~30 nm thick) and GaAs_0.46_P_0.54_ barriers (~19 nm thick). Note that the relative phosphorous concentrations are increased (~10%) from their nominal values to achieve good agreement with experimental data, as discussed in the main text. Various direct and indirect absorption transition wavelengths were determined by the position of the bandedges (*Γ* and *X* conduction bands; *HH* and *LH* valence bands) within the core and barriers of the structure. See Supplementary Information for the calculated bandedge profile throughout the heterostructure.

### i-TCSPC measurements

The emission from the μ-PL set-up was coupled into the i-TCSPC system via a single-mode fibre (Thorlabs 780HP). The i-TCSPC system consists of a two-channel folded Michelson interferometer driven by a closed-loop piezo translation stage with two silicon single-photon avalanche photodiode detectors. A synchronisation signal was obtained from the excitation laser and connected to HydraHarp 400 TCSPC hardware. Photon counts were kept below 5% of the laser repetition rate to avoid photon pile-up. The delay stage performed bidirectional periodic motion at a frequency of 1.0 Hz across the position of the zero optical delay, and photon arrivals were recorded. i-TCSPC measurements were taken on ten wires at various powers along the threshold curves with ~30 × 10^6^ photons recorded per scan. The i-TCSPC measurements yielded similar behaviour for all NWs, and the results reported here are restricted to one typical wire. See Supplementary Information and a recent similar technique demonstration^48^ for more details of the i-TCSPC set-up and principals of operation.

### Transmission electron microscopy

The scanning TEM (STEM) images shown in Fig. 1d and in the Supplementary Information were obtained using a doubly corrected ARM200F microscope operating at 200 kV. The cross-sections of the NWs were prepared by embedding them in a low-viscosity resin and slicing using a microtome. See Supplementary Information for additional TEM images of the quantum wells and filtered planes.

## Supplementary information

Supplemental Material
