# Supplementary material for: Heterostructure and Q-factor engineering for low-threshold and persistent nanowire lasing
Source: Light Sci Appl. 2020 Mar 17;9:43. doi: 10.1038/s41377-020-0279-y (PMC7078256; doi:10.1038/s41377-020-0279-y)
Supplement: Supplementary file 1 — Supplemental Material [file 41377_2020_279_MOESM1_ESM.pdf]

Supplementary Information: Heterostructure and Q-factor Engineering for Low-threshold and Persistent Nanowire Lasing

Stefan Skalsky,[1] Yunyan Zhang,[2] Juan Arturo Alanis,[1] H. Aruni Fonseka,[3] Ana M. Sanchez,[3] Huiyun Liu,[2] and Patrick Parkinson[1]

[1]Department of Physics and Astronomy, The Photon Science Institute, The University of Manchester, Manchester, M13 9PL, United Kingdom
[2]Department of Electronic and Electrical Engineering, University College London, London, WC1E 7JE, United Kingdom
[3]Department of Physics, University of Warwick, Coventry, CV4 7AL, United Kingdom

I. SCANNING TRANSMISSION ELECTRON MICROSCOPY (STEM) IMAGING

Cross-sectional STEM imagery was acquired using a doubly corrected ARM200F microscope at 200 kV, as described in the main text. The microscopy was analysed along three {220} planes as shown in Figure S1. Any misfit dislocation formed between the quantum well (QW) and barrier would appear as a line that is present on one but not both sides of the interface (for a missing plane within one region). The absence of such lines across the interface supports the high quality of the QW region.

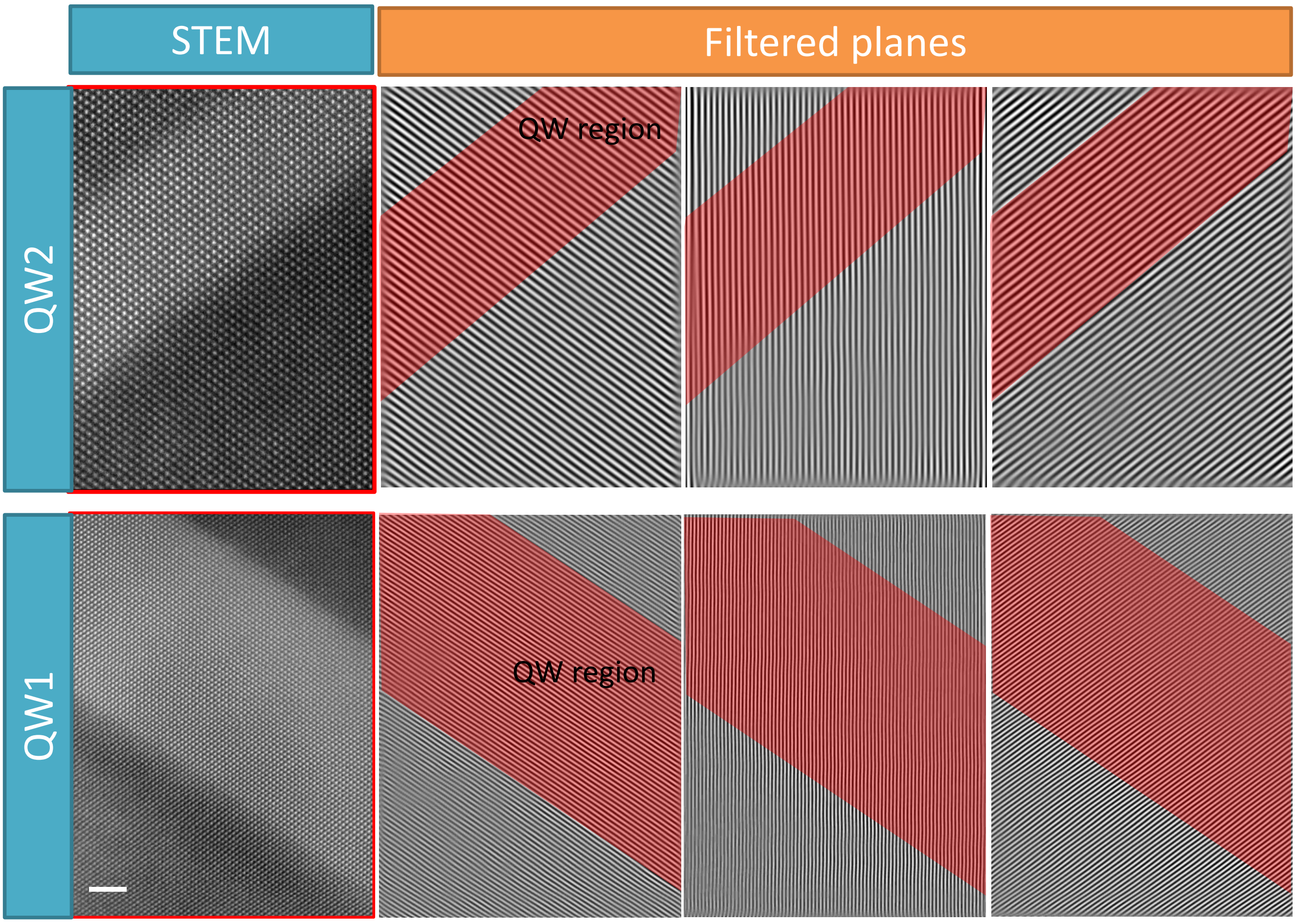

Figure S1: Annular dark field (ADF) scanning transmission electron microscope (STEM) images of two quantum wells (QWs) within the same NW and corresponding fast Fourier transform (FFT) filtered images. The lines in the three respective filtered images correspond to the 220 planes in the three directions on the (111) plane. Scale bar represents 2 nm.

II. LASING DATA FOR ADDITIONAL NANOWIRES

A series of emission spectra and integrated emission as a function of excitation fluence are shown for 18 additional randomly selected example NW lasers in Figure S2 illustrating typical lasing characteristics under 620 nm excitation. These demonstrate the range of thresholds, lasing wavelengths and slope efficiency within the sample sets. It is noted that near-linear threshold behaviour is commonly observed, along with frequent single mode emission. Along with the observation of a range of lasing wavelengths, these findings support the premise of high quality laser cavities with inhomogeneous and potentially non-optimal quantum well growth.

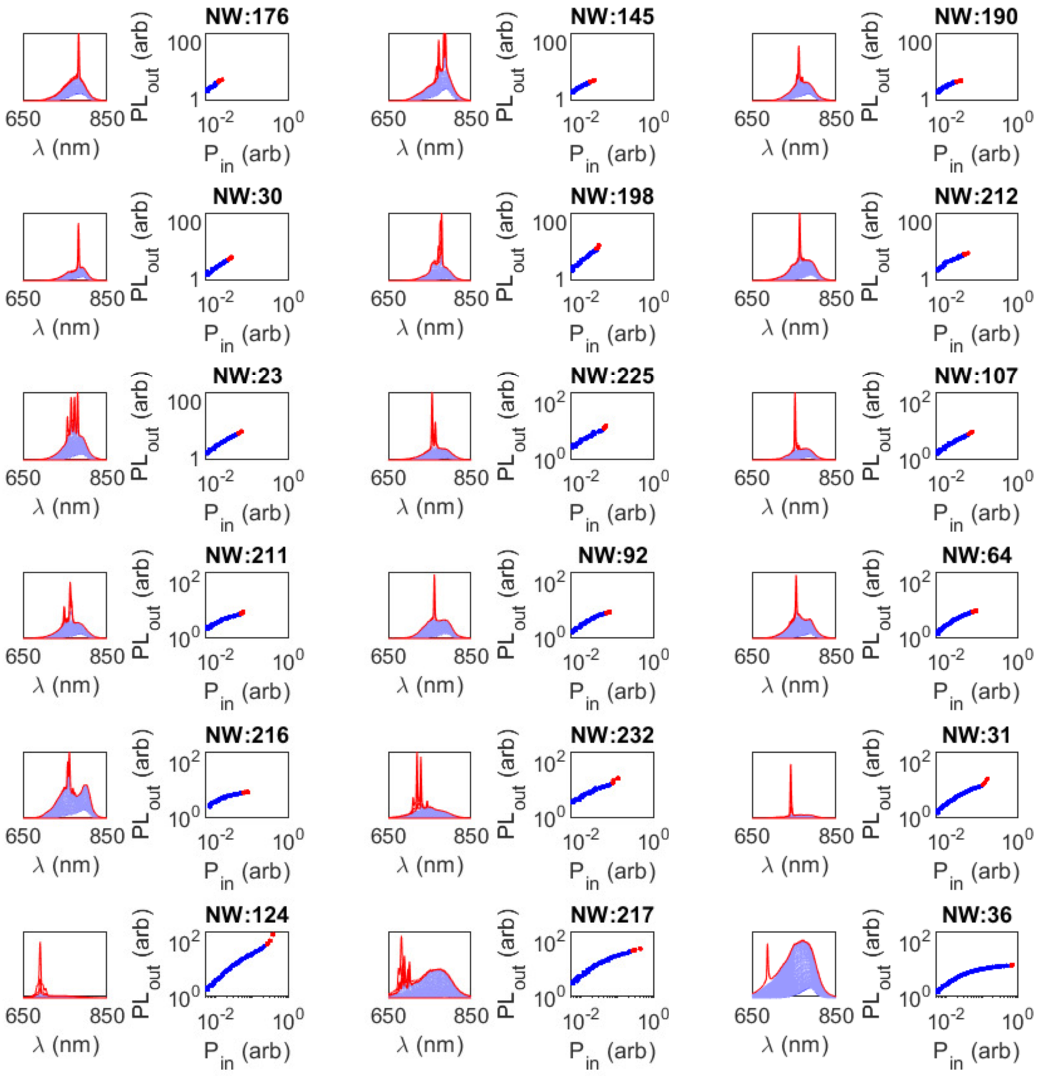

Figure S2: A series of spectra (left) and PL intensity as a function of excitation power (right) for 18 randomly selected NWs. Points and spectra in blue are before lasing is identified; points and spectra in red are above lasing threshold.

III. DAMAGE THRESHOLD MEASUREMENTS

The ratio of the lasing threshold to the damage threshold is a critical parameter determining lasing yield. Characterisation of the NW laser damage resistance was studied *via* power-dependent excitation measurements on ten NLs. The excitation intensity was controlled *via* rotation of a neutral density filter wheel through minimum to maximum intensity available. The measured output emission intensity was recorded as a function of excitation fluence to determine the point at which the output intensity drops. An example of such a plot is shown in Figure S3 where the damage threshold ($D_{\rm th}$) is reached at $\sim 2400\mu$Jcm^{-2}pulse^{-1}; this is at a fluence of $\sim$10$\times$ the lasing threshold ($L_{\rm th}\sim$ 240μJcm^{-2}pulse^{-1}).

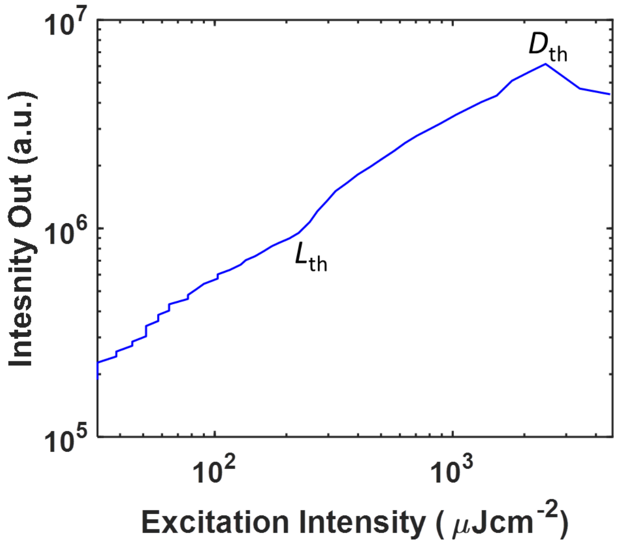

Figure S3: Damage threshold ($D_{\rm th}$) measured for an example NL under 620 nm excitation. For this wire, $D_{\rm th}$ occurs at an excitation intensity $\sim$10$\times L_{\rm th}$.

IV. PHOTOLUMINESCENCE EXCITATION (PLE) MEASUREMENT FOR ALL NWS

The normalized PLE spectra for all seven studied NWs are shown Figure S4. The high resolution measurement (bottom spectra) is the example displayed in the main text. Very similar behaviour is displayed for all wires demonstrating consistency, although differences in the relative height and centre positions of the three peaks (530 nm, 570 nm and 610 nm) are observed.

V. CHAMPION THRESHOLD MEASUREMENT

Under 570 nm excitation we demonstrate record low thresholds for III-V semiconductor NW lasers. The power dependent spectra and resulting intensity-in vs intensity-out curve for this champion NL are shown in Figure S5. Here emission intensity output is integrated over all wavelengths and the behaviour appears near-linear indicating that this may be a high β wire.

VI. INTERFEROMETRIC TIME CORRELATED SINGLE PHOTON COUNTING (I-TCSPC)

i-TCSPC is a technique which combines Fourier transform spectroscopy and TCSPC to obtain ultra-sensitive simultaneous measurements of time and spectrally resolved emission. Our setup is shown in the schematic in Figure S6 and consists of a two-channel folded Michelson interferometer with the movable retro-reflector (RR) driven by a closed-loop piezo translation stage. The detectors are of the form of silicon single photon avalanche photodiodes (SPADs) connected to TCSPC hardware (HydraHarp 400) which receive a synchronisation signal from the excitation

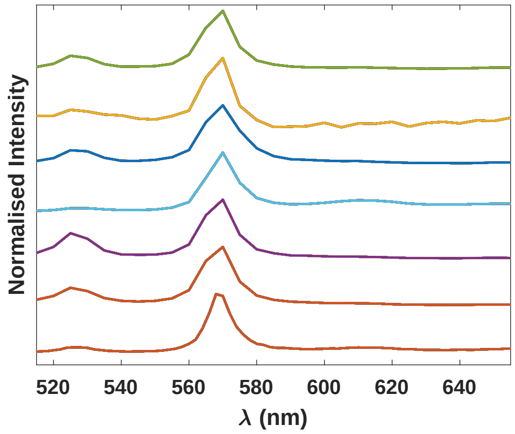

Figure S4: Stacked normalized PLE spectra for seven studied NWs.

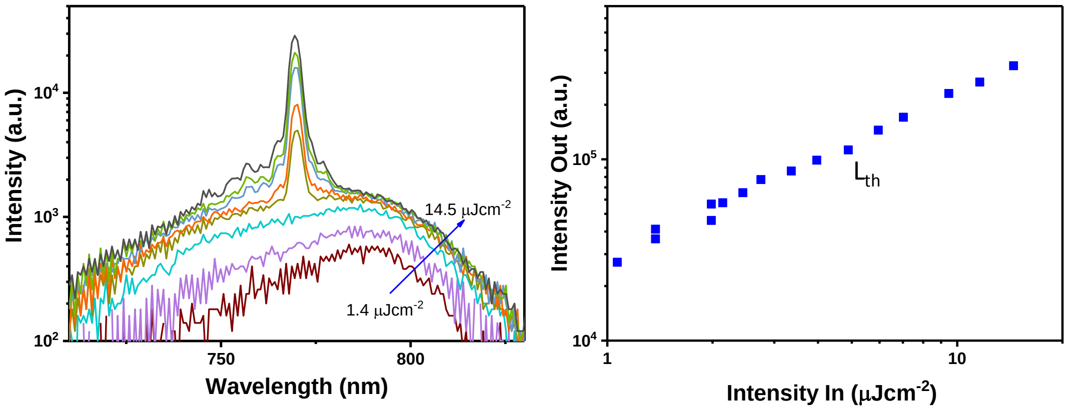

Figure S5: (a) Power dependent spectra on log scale and (b) resulting intensity-in vs intensity-out curve on log-log scale for a selected NW laser under 570 nm excitation. A threshold of $\sim 6\mu\mathrm{Jcm}^{-2}\mathrm{pulse}^{-1}$(marked L_{th}) is measured.

laser enabling time-resolved measurements. The sample emission from the μ-PL setup is fed into the interferometer *via* a single mode fibre and collimated. The measurement process involves scanning the movable mirror (RR2) through the position of zero-delay (δ=0) and storing a record of each photon arrival consisting of the delay-stage position (δ), time of arrival since excitation (τ) and channel of photon arrival. This information enables measurement of the emission interferogram as well as time-decay. By taking the Fourier transform of the interferogram for photon arrivals within τ windows along the decay, time-resolved PL maps can be obtained (as seen in Figure 4 of the main text). The use of two channels in a folded setup enables throughput and differential interference enhancement (through subtraction of the out-of-phase channel signals).

VII. COHERENCE LENGTH MEASUREMENT AND CAVITY CALCULATIONS

The coherence length of the NLs exceeded the maximum optical delay provided by the interferometer delay-stage (200 μm). However the fixed mirror in the setup was mounted on a mechanical translation stage capable of providing up to 6 mm of delay with 5 μm precision. In order to recover the interference pattern to measure the lasing coherence length, a procedure consisting of a 200 μm i-TCSPC scan at δ=0 followed by successive 50 μm fixed mirror translations (providing 100 μm delay) and repeated measurement was used. Measurements after each fixed mirror translation were carried out under the same excitation conditions (time and power). The long-range NL interferogram was then recovered from a stitching method in post processing. Since the interference pattern is symmetrical through δ=0 μm, fixed mirror translation in only one direction was required. By reversing the data in the appropriate positions a bi-directional interferogram was recovered. By carrying out a fit to the interference visibility ($\frac{I_{\max}(\delta)-I_{\min}(\delta)}{I_{\max}(\delta)+I_{\min}(\delta)}$) with

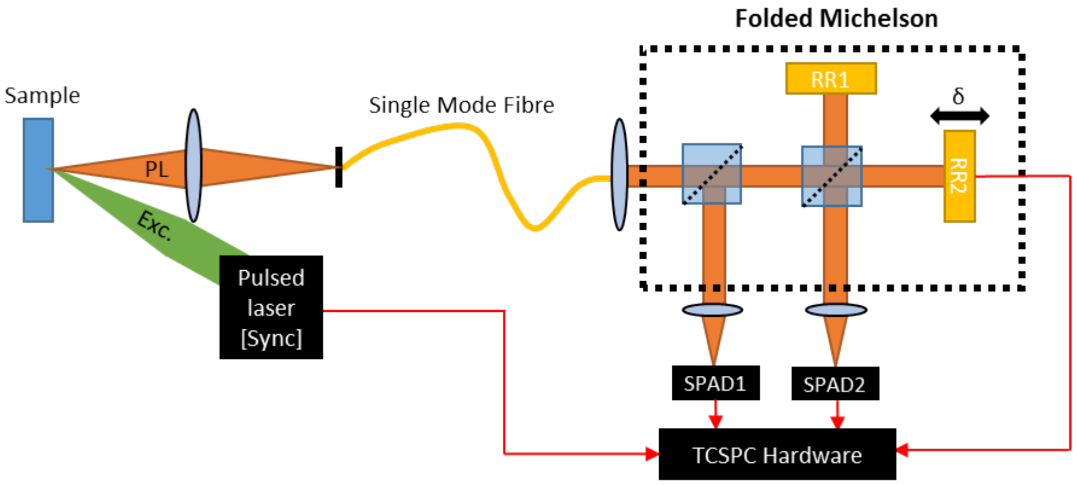

Figure S6: Schematic of i-TCSPC setup; a folded two channel closed-loop single-photon sensitive interferometer is shown inside the dashed black box. Emission input is fibre-coupled from the μ-PL setup. Electrical signals are shown as red lines.

Gaussian PL and lasing components the coherence length could be estimated from the FWHM of the lasing fit. Figure S7 shows the bi-directional recovered interferogram (a) and interference visibility and fit (b).

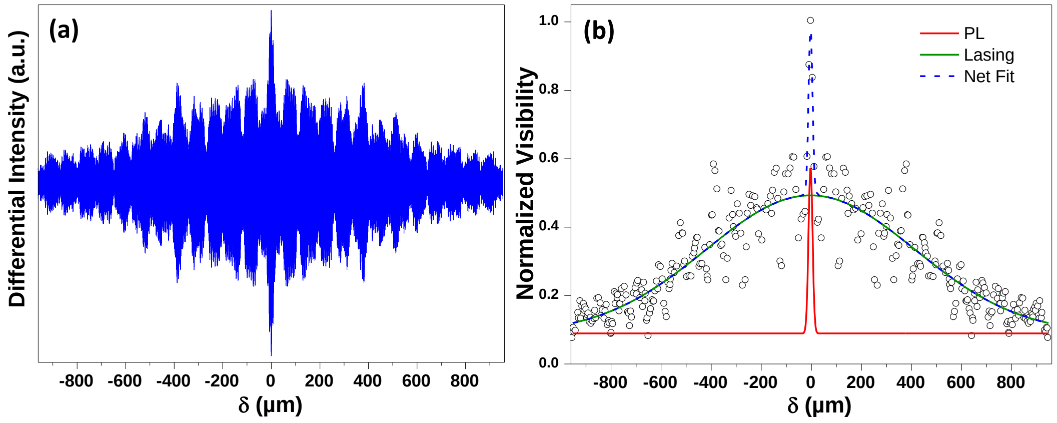

Figure S7: Numerically reflected bi-directional interferogram produced by exploiting the symmetry of the system (a) and interference visibility and fit (b).

The PL component FWHM ($\sim$18 μm) is similar for the interference visibility fit determined just below threshold where PL clamping occurs ensuring these values are meaningful. $L_{\mathrm{coh}} = 980 \pm 70\,\mu$m was obtained with error propagation of the stage translation error and standard error on the fit. Note only the real half of the data is displayed in the main text. The standard laser cavity equations used for visibility and cavity Q-factor and reflectivity (R) calculations are shown below where τ_{coh} and τ_{p} are the coherence time and cavity lifetime respectively. Here we consider the end-facet reflectivity to be $R = \sqrt{R_1 R_2}$ and used a NW length L= 14.5 μm deduced from an optical image of the NW in the PL regime (where the centre of emission points are taken to be the end facet positions). A GaAsP refractive index (n) of 3.4 at λ_{Las} =780 nm was estimated from literature values for GaAsP[1].

Coherence Time $$\tau_{\mathrm{coh}} = \frac{L_{\mathrm{coh}}}{c}$$

Cavity Lifetime $$\tau_{\mathrm{p}} = \frac{\tau_{\mathrm{coh}}}{2\pi}$$

Cavity Lifetime $$\tau_{\mathrm{p}} = \frac{2nL/c}{\ln\left(1/R_1 R_2\right)}$$

Cavity Q-Factor $$Q = \omega \tau_{\mathrm{p}}$$

VIII. TIME-RESOLVED SPECTRAL ANALYSIS

Time-resolved spectral maps shown in Figure 4 of the main text were produced by taking the Fourier transform of the interferograms generated by photon arrivals in time-gated windows and taking the normalized absolute value of the resulting time-gated spectra. Analysis of the relative lasing/PL intensities was performed by carrying out fits of these spectra with Gaussian PL and lasing components (described by Equation 1 in main text). The relative peak area magnitudes $A_{\mathrm{Las}}/A_{\mathrm{PL}}$ and peak PL energies (E_{PL}) were recorded and studied as a function of excitation power and time since excitation (τ) to gain insight into the carrier/photon dynamics and underlying nanolasing mechanism.

IX. NEXTNANO MODELLING

One-dimensional simulations under pseudomorphic strain conditions were performed in nextnano software, to determine the band-edge positions throughout the heterostructure where absorption transitions may occur. The band-edges of the Γ and X conduction bands and the HH and LH valence band are shown in Figure S8 as a function of radial distance from the core. The magnitude of confinement for holes in the valence band quantum well is atypically larger than that of the electrons within the conduction band. This is known to occur due to strain related effects[2]. It is also notable that the light-hole heavy-hole degeneracy is lifted in the barrier due to the applied strain.

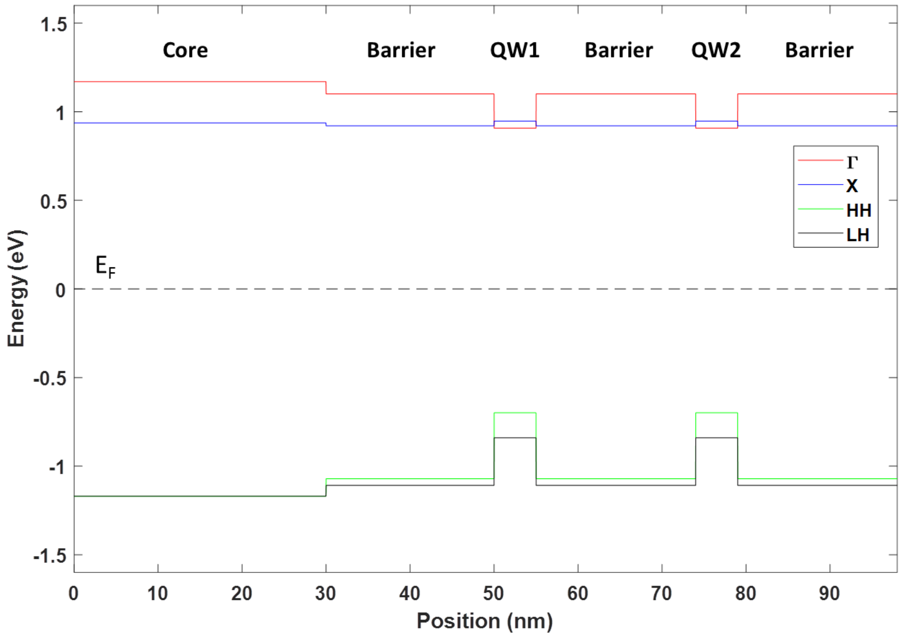

Figure S8: Calculated band-edge positions within the heterostructure with Fermi level (E_{F}) set to be the zero energy reference. Importantly the X conduction band is near isoenergetic (within k_{B}T) throughout the structure enabling carrier transfer from barriers to quantum wells. The LH and HH band-edge energies are within close proximity throughout the structure.

[1] G. D. Clark and N. Holonyak, Physical Review **156**, 913 (1967).
[2] H. A. Fonseka, A. V. Velichko, Y. Zhang, J. A. Gott, G. D. Davis, R. Beanland, H. Liu, D. J. Mowbray, and A. M. Sanchez, Nano Letters **19**, 4158 (2019), ISSN 1530-6984, URL `http://pubs.acs.org/doi/10.1021/acs.nanolett.9b01673`.
